# Engineering a Culturable Serratia symbiotica Strain for Aphid Paratransgenesis

**DOI:** 10.1128/AEM.02245-20

**Published:** 2021-01-29

**Authors:** Katherine M. Elston, Julie Perreau, Gerald P. Maeda, Nancy A. Moran, Jeffrey E. Barrick

**Affiliations:** a Department of Molecular Biosciences, The University of Texas at Austin, Austin, Texas, USA; b Department of Integrative Biology, The University of Texas at Austin, Austin, Texas, USA; University of Queensland

**Keywords:** endosymbionts, paratransgenesis, synthetic biology

## Abstract

Aphids are global agricultural pests and important models for bacterial symbiosis. To date, none of the native symbionts of aphids have been genetically manipulated, which limits our understanding of how they interact with their hosts. Serratia symbiotica CWBI-2.3^T^ is a culturable, gut-associated bacterium isolated from the black bean aphid. Closely related Serratia symbiotica strains are facultative aphid endosymbionts that are vertically transmitted from mother to offspring during embryogenesis. We demonstrate that CWBI-2.3^T^ can be genetically engineered using a variety of techniques, plasmids, and gene expression parts. Then, we use fluorescent protein expression to track the dynamics with which CWBI-2.3^T^ colonizes the guts of multiple aphid species, and we measure how this bacterium affects aphid fitness. Finally, we show that we can induce heterologous gene expression from engineered CWBI-2.3^T^ in living aphids. These results inform the development of CWBI-2.3^T^ for aphid paratransgenesis, which could be used to study aphid biology and to enable future agricultural technologies.

**IMPORTANCE** Insects have remarkably diverse and integral roles in global ecosystems. Many harbor symbiotic bacteria, but very few of these bacteria have been genetically engineered. Aphids are major agricultural pests and an important model system for the study of symbiosis. This work describes methods for engineering a culturable aphid symbiont, Serratia symbiotica CWBI-2.3^T^. These approaches and genetic tools could be used in the future to implement new paradigms for the biological study and control of aphids.

## Abstract (precis)

Insects have remarkably diverse and integral roles in global ecosystems. Many harbor symbiotic bacteria, but very few of these bacteria have been genetically engineered.

## INTRODUCTION

Many insects have a characteristic bacterial microbiome. These associations can take many different forms, ranging from the conserved gut communities of bees (1) to symbionts of sap-sucking insects that have evolved to resemble organelles (2). These and other bacterium-host relationships have inspired attempts to study and to control insects by genetically engineering their resident microbiomes. This approach, known as “paratransgenesis,” has been developed primarily for insects that are vectors of human disease, including kissing bugs, tsetse flies, and mosquitoes (3–5). Recently, the feasibility of paratransgenesis has also been demonstrated in agricultural pests, where it could provide an alternative to chemical pesticides and the development of genetically engineered crops (6–9).

Aphids are major worldwide agricultural pests and vectors for many plant viruses (10–12). They are also model organisms for understanding insect-endosymbiont coevolution because they have evolved close relationships with multiple species of bacterial symbionts. Typically, aphid symbionts are housed in specialized host cells called bacteriocytes and are reliably vertically transmitted from mother to offspring (2, 13). The obligate symbiont Buchnera aphidicola plays an essential role in producing nutrients lacking in the aphid diet. The relationship between *Buchnera* and its host epitomizes a common dynamic in natural symbioses, in which bacteria and host are completely dependent on one another for survival. Aphids can also be associated with facultative symbionts such as “*Candidatus* Hamiltonella defensa,” “*Candidatus* Regiella insecticola,” or Serratia symbiotica, which provide benefits in stressful environments (14). These nonessential symbionts have more complex relationships with their aphid hosts (15). “*Candidatus* Hamiltonella defensa” protects aphids from wasp parasitism, but it also reduces host reproduction and longevity (16–18). Serratia symbiotica can impose fitness costs on aphids that include increasing their susceptibility to insecticides, but it has also been shown to improve host survival in response to heat stress and wasp parasitism (19–23).

In 2011, a culturable strain of S. symbiotica, CWBI-2.3^T^, was isolated from the black bean aphid, Aphis fabae (24). CWBI-2.3^T^ is a member of a widespread S. symbiotica clade that is distinct from the bacteriocyte-associated symbiont strains. These gut-associated S. symbiotica strains appear to be at a primitive or transitional stage of symbiosis with aphids (23, 25). More specialized facultative symbiont strains of S. symbiotica, such as Tucson and IS, are found primarily in bacteriocytes and the insect hemolymph and are faithfully transmitted to all of a colonized mother’s offspring (26). In contrast, S. symbiotica CWBI-2.3^T^ primarily colonizes the digestive tracts of aphids and exhibits only sporadic transmission to progeny (23, 27). Support for CWBI-2.3^T^ resembling a transitional symbiont also comes from its genome, which is intermediate in size between genomes of the strictly symbiotic strains and genomes of related free-living *Serratia* species (28, 29). CWBI-2.3^T^ is thought to spread in natural populations of aphids through two horizontal transmission routes. It is excreted in aphid honeydew (liquid feces) onto plant surfaces, which could lead to environmental transmission (23, 25, 30), and it has been reported to spread between aphids feeding on the same plant by colonizing the phloem (31).

We examined the potential of Serratia symbiotica CWBI-2.3^T^ for paratransgenesis in aphids. We found that many plasmids, gene expression parts, and techniques used in Escherichia coli function in CWBI-2.3^T^. We used a fluorescently labeled strain to show that S. symbiotica CWBI-2.3^T^ can be reliably established in the guts of multiple species of aphids by feeding. However, colonization with CWBI-2.3^T^ eventually leads to decreased survival of some species. Finally, we show that we can achieve inducible expression of green fluorescent protein (GFP) in engineered CWBI-2.3^T^ living inside the aphid gut. No other aphid symbionts have been genetically engineered to date. Thus, CWBI-2.3^T^ represents a promising chassis organism for achieving short-term paratransgenesis, as well as an intriguing organism in which the genetic approaches that we describe could be used to study evolutionary transitions from insect pathogens to symbionts.

## RESULTS

### Genetic engineering of S. symbiotica CWBI-2.3^T^.

We first tested whether common genetic techniques and DNA parts functioned in S. symbiotica CWBI-2.3^T^. We began by measuring the growth rate of the wild-type strain. Its doubling time in culture is approximately 4 h. Accounting for this slower growth rate allowed for the successful transformation of CWBI-2.3^T^ through conjugation and electroporation procedures used in E. coli. By conjugating a nonreplicating donor plasmid encoding a mini-Tn*7* construct from E. coli into S. symbiotica CWBI-2.3^T^, we were also able to integrate GFP into a specific location in its chromosome. Integration of engineered constructs in this way enables their functions to be stably maintained without the need for antibiotic selection (32). The resulting GFP-expressing S. symbiotica CWBI-2.3^T^ strain (CWBI-2.3^T^-GFP) can be used to monitor the colonization of insects, as we show below.

**FIG 1:**
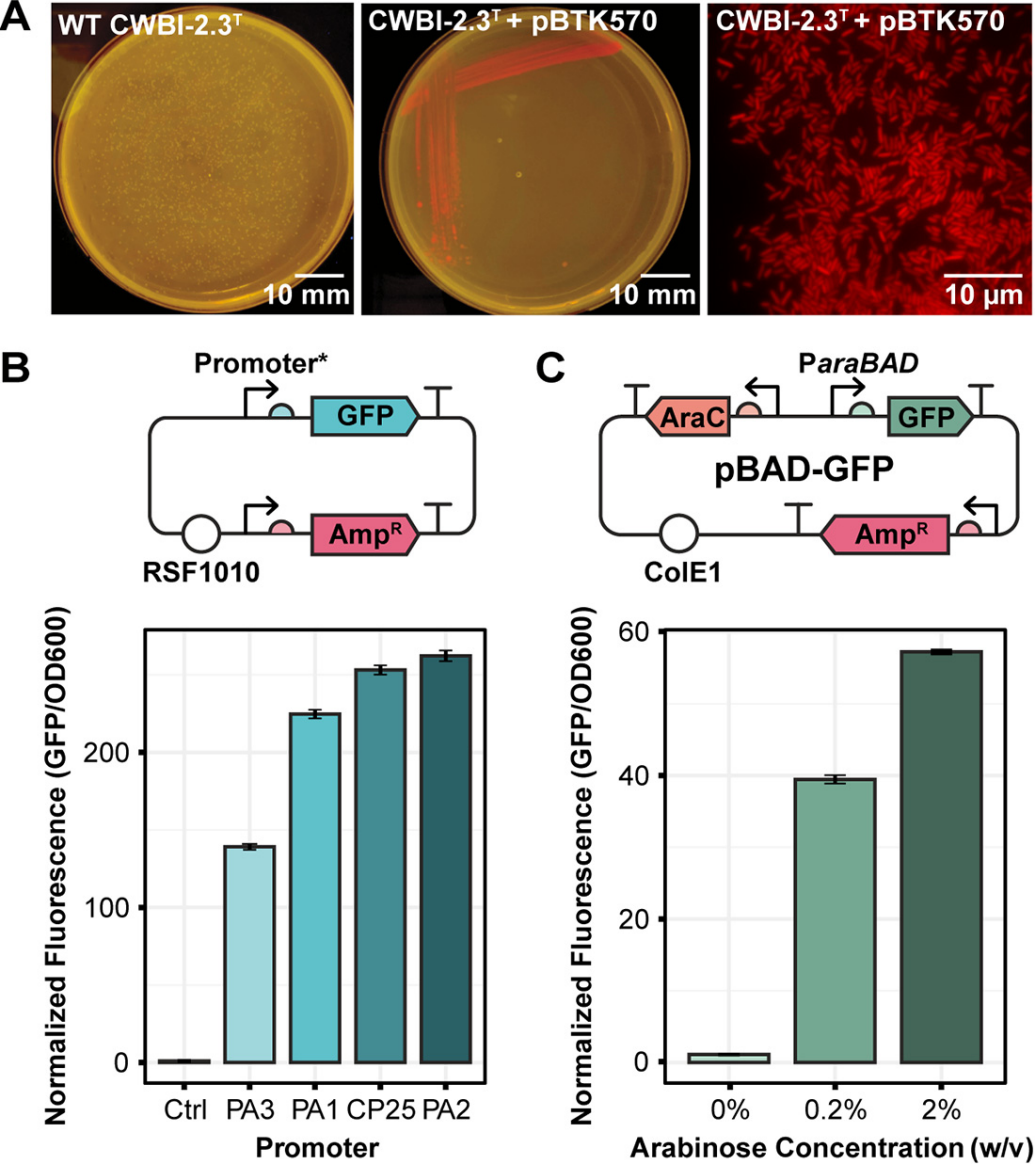
Origin and promoter function in Serratia symbiotica CWBI-2.3^T^. (A) Representative images of CWBI-2.3^T^ transformed with an RSF1010-origin plasmid expressing E2 Crimson. The left two images comparing this strain to wild-type CWBI-2.3^T^ were captured at the macroscopic scale using a blue light transilluminator and were edited with linear adjustments. The image on the right was captured at the microscopic scale using a Nikon Eclipse inverted fluorescence microscope (excitation at 640 nm and emission at 685 nm) and was linearly adjusted and pseudocolored. Plasmid features in panels B and C are shown using Synthetic Biology Open Language Visual symbols: promoter (bent arrow), ribosome binding site (semicircle), open reading frame (block arrow), terminator (T bar), and origin of replication (open circle). (B) Normalized expression of GFP from a series of plasmids that are identical except for their GFP promoter sequences. The plasmid map is shown above, and expression levels are shown below. Expression for each promoter is normalized to the GFP/OD_600_ reading for wild-type CWBI-2.3^T^ (Ctrl). (C) Normalized expression of GFP from CWBI-2.3^T^-pBAD-GFP following induction with arabinose. A schematic of pBAD-GFP is shown above, and expression levels are shown below. Fluorescence at each inducer concentration is normalized to the GFP/OD_600_ reading for wild-type CWBI-2.3^T^.

We also screened CWBI-2.3^T^ for its ability to maintain plasmids containing different origins of replication and antibiotic resistance cassettes, some of which have been reported to function in Serratia marcescens strains (33–36). We transformed plasmids that had either broad-host-range or E. coli-specific origins, as well as different copy numbers. Additionally, we tested whether CWBI-2.3^T^ was compatible with several common antibiotic resistance genes. We achieved successful transformants for every plasmid and antibiotic tested (Table 1). CWBI-2.3^T^ transformed with pBTK570, an RSF1010 plasmid with spectinomycin resistance that expresses E2 Crimson, is shown in Fig. 1A. These results demonstrate great flexibility in how CWBI-2.3^T^ can be genetically modified using combinations of plasmids and genome integration.

**TABLE 1:** Plasmid origins and antibiotic resistance genes that function in S. symbiotica CWBI-2.3^T^

Origin or resistance	Copy number	Host range	Resistance gene	Plasmid(s) or construct(s)	Reference(s)
Origin					
RSF1010	Low	Broad		pBTK570, pBTK501, pBTK503, pBTK509, pBTK510	36
p15A	Low	Narrow		pBTK409	36
pBBR1	Medium	Broad		pAK*gfplux*1	50
ColE1	High	Narrow		pYTK001, mTagBFP2-pBAD	48, 51
Antibiotic resistance					
Ampicillin/carbenicillin			*bla*_TEM-116_	pAK*gfplux*1, pBTK409, pBTK501, pBTK503, pBTK509, pBTK510	36, 50
Ampicillin/carbenicillin			*bla*_TEM-181_	mTagBFP2-pBAD	48
Chloramphenicol			*catI*	pYTK001	51
Gentamicin			*aacC1*	pBT20	52, 53
Kanamycin/neomycin			*aphA-2* (*nptII*)	pTn*7*-PA1-GFP-kan	32, 36
Spectinomycin			*aadA*	pBTK570	36

To complete our assessment of the capabilities of S. symbiotica CWBI-2.3^T^, we tested the performance of different constitutive and inducible promoters. For applications requiring continuous gene expression, we examined the relative levels of GFP expression from four broad-host-range constitutive promoters, namely, PA1, PA2, PA3, and CP25 (36). Each of these promoters was functional, yielding a fluorescent signal 140 to 260 times brighter than the background autofluorescence of the wild-type strain (Fig. 1B). To allow for controlled gene expression, we also tested the functionality of the pBAD plasmid system, which uses the *araC* regulator to enable arabinose-inducible expression from the *araBAD* promoter. We found that this system behaved as expected. There were low levels of GFP expression in the absence of arabinose: the average fluorescence of the uninduced strain containing the pBAD-GFP plasmid was only 1.12 times the background level of wild-type CWBI-2.3^T^ with no plasmid. GFP fluorescence in the strain with the pBAD-GFP plasmid became 39 and 57 times brighter than that in the wild-type strain when induced with 0.2% and 2% arabinose, respectively (Fig. 1C).

### Engineered S. symbiotica CWBI-2.3^T^ can colonize multiple species of aphids.

To test the versatility of engineered CWBI-2.3^T^ for paratransgenesis, we assessed its ability to colonize multiple species of aphids and monitored the colonization dynamics using CWBI-2.3^T^-GFP. We first established a method for delivering engineered CWBI-2.3^T^ to aphids by adapting the methods of Renoz et al. (30). CWBI-2.3^T^-GFP was delivered to third instar aphids through feeding on an artificial diet. Three days after feeding, a blue light transilluminator could be used to observe GFP fluorescence from CWBI-2.3^T^-GFP inside the colonized aphids. Using this feeding technique with Acyrthosiphon pisum, we were able to reliably colonize an average of about 75% of treated aphids.

**FIG 2:**
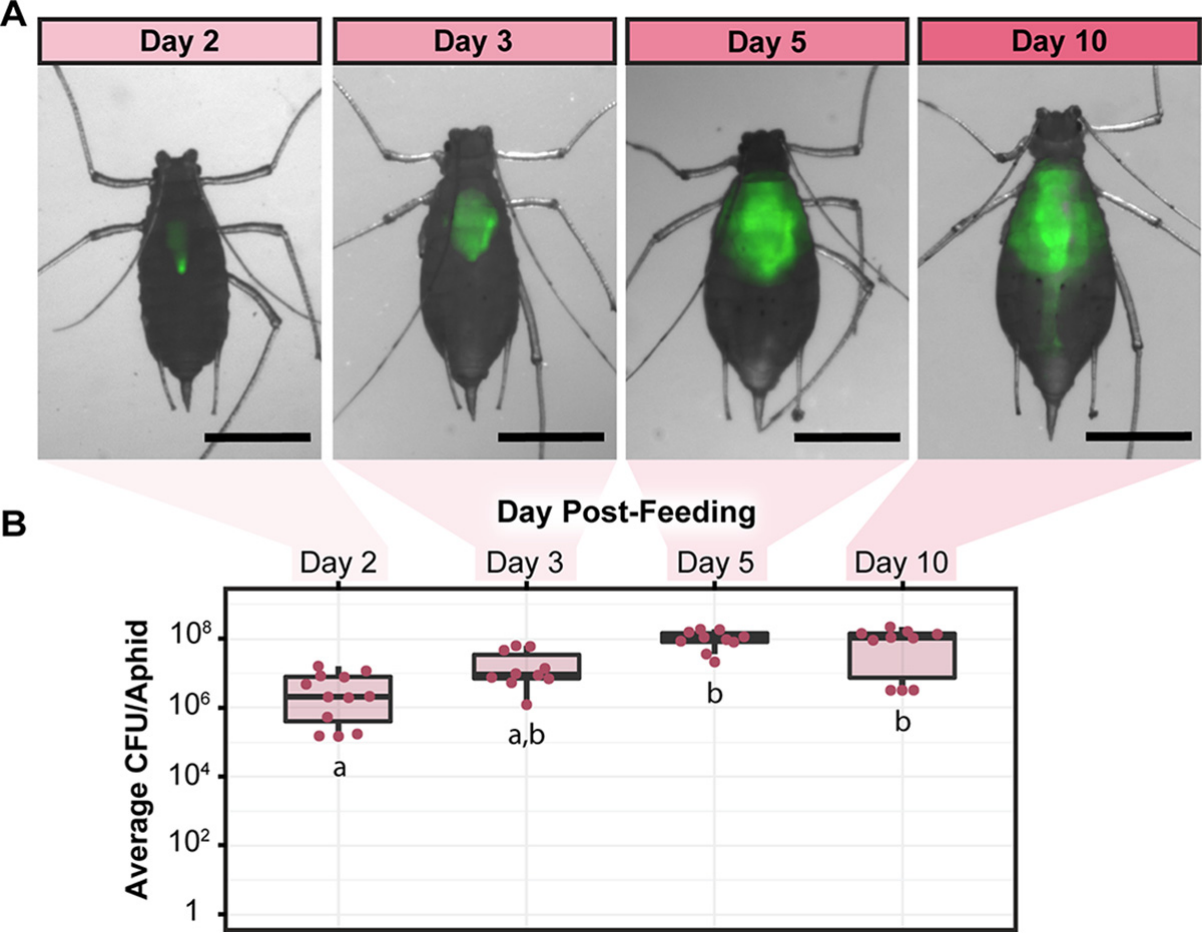
Colonization dynamics of CWBI-2.3^T^ in A. pisum. (A) Composite brightfield and fluorescence images tracking the spread of CWBI-2.3^T^-GFP through the gut of a live A. pisum individual. Dorsal images of the same aphid were captured for each time point. Images were linearly adjusted to enhance the brightness of GFP. The scale bar is 1 mm. (B) CWBI-2.3^T^-GFP titer in A. pisum over time. At least 10 aphids per time point were crushed and plated to perform CFU counts. Lowercase letters (a and b) below the bars designate groups of time points at which significantly different colonization levels were observed (*P < *0.05, Dunn's test with Bonferroni correction).

**FIG 3:**
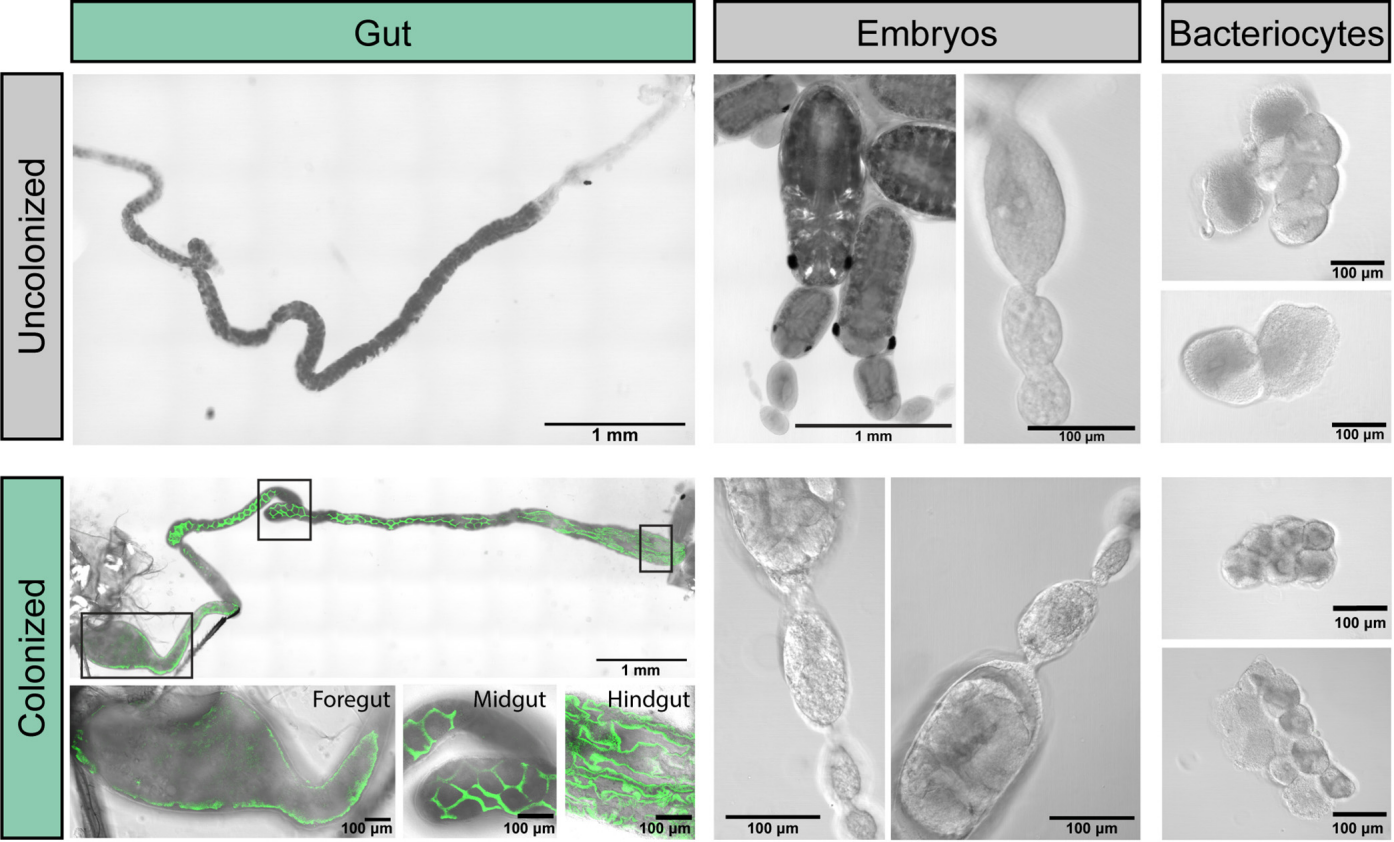
CWBI-2.3^T^-GFP colonization of the aphid gut. A. pisum aphids fed on diet alone (uncolonized) and diet containing CWBI-2.3^T^-GFP (colonized) were dissected at 10 days following treatment. Laser scanning confocal microscopy was used to capture brightfield and GFP images for each tissue type, and images were linearly adjusted to enhance the brightness of GFP. CWBI-2.3^T^-GFP was observed only in the aphid gut and not in embryos, bacteriocytes, or other tissues.

The fluorescence of the CWBI-2.3^T^-GFP strain also allowed us to monitor how CWBI-2.3^T^ colonizes living aphids. We observed that over the course of 10 days S. symbiotica CWBI-2.3^T^ spread through the digestive tract of A. pisum until the entire gut was colonized (Fig. 2A and Fig. 3). No bacteria were observed outside the gut in bacteriocytes or embryos (Fig. 3), and the gut remained colonized and fluorescent for the duration of experiments (up to 10 days after feeding for A. pisum). We also quantified host colonization by tracking the number of CFU per aphid. On 2, 3, 5, and 10 days postfeeding, colonized A. pisum aphids were crushed and plated to determine the bacterial loads in their bodies. The CWBI-2.3^T^ titer increased over time until it reached an average of about 10^8^ CFU per aphid on day 5. Between day 5 and day 10, no significant change in titer was observed (*P = *0.80, two-tailed Mann-Whitney *U* test) (Fig. 2B). All of these results are consistent with prior studies that used fluorescence *in situ* hybridization and quantitative PCR to monitor A. pisum colonization (30).

**FIG 4:**
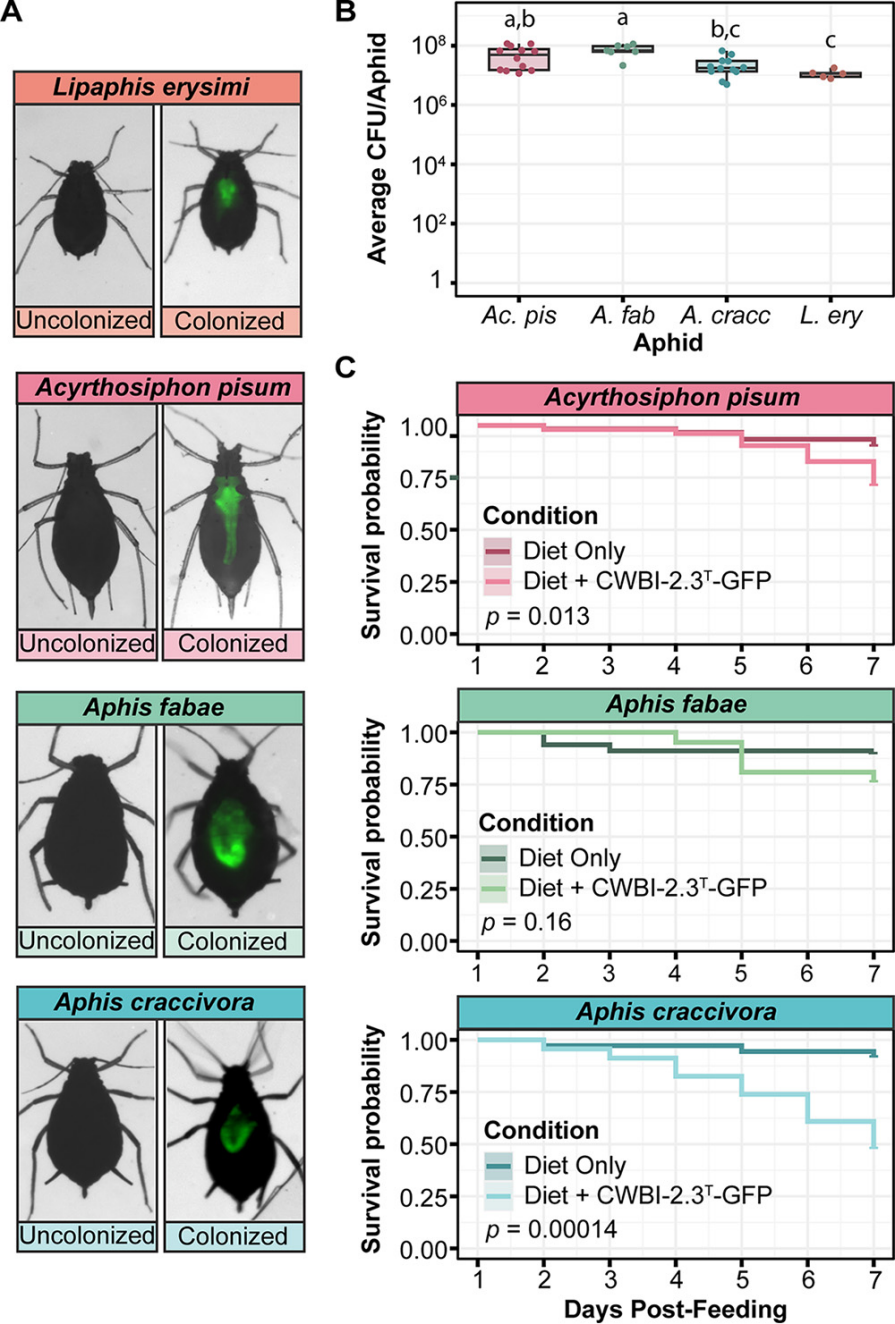
CWBI-2.3^T^-GFP colonization of multiple aphid species. (A) Composite brightfield and fluorescent stereoscope images showing CWBI-2.3^T^-GFP colonization of four different species. Photographs for the uncolonized and colonized pairs were captured with the same exposure for comparison. Images were captured from the ventral side of each aphid on the 5th day after feeding. (B) CWBI-2.3^T^-GFP titer in each of the four species on day 5 postfeeding. At least five aphids per species were crushed and plated to perform CFU counts. Lowercase letters (a, b, and c) above the bars designate sets of aphid species for which significantly different colonization levels were observed (*P < *0.05, Dunn's test with Bonferroni correction). (C) Mortality curves showing the survival probability of each aphid colonized with CWBI-2.3^T^-GFP, compared to uncolonized aphids. Counts began on the first day after feeding to eliminate aphids that died during experimental setup. The starting population was at least 21 aphids for each survival curve. The statistical significance of differences in aphid survival was calculated using a log-rank test (*P* values are shown in each panel).

We next tested whether the colonization pattern in A. pisum was representative of that in other aphid species by repeating this experiment with Aphis fabae (black bean aphid), Aphis craccivora (cowpea aphid), and Lipaphis erysimi (mustard aphid). We found that CWBI-2.3^T^-GFP principally colonized the guts of these three species as well (Fig. 4A). Each of the other aphids tested also showed a similar trend in bacterial titers over time. The CFU per aphid values consistently reached between 10^7^ and 10^8^ CFU per aphid on the fifth day after treatment for all four species, with averages of 5.3 × 10^7^ CFU per aphid, 7.4 × 10^7^ CFU per aphid, 2.4 × 10^7^ CFU per aphid, and 1.2 × 10^7^ CFU per aphid for A. pisum, A. fabae, A. craccivora, and L. erysimi, respectively (Fig. 4B). Slight but significant differences in bacterial titers were observed in the four species (*P = *0.0018, Kruskal-Wallis test). The titer was highest in A. fabae, which is the natural host from which CWBI-2.3^T^ was isolated. The trend in the other species may reflect their different body sizes, as A. pisum is much larger than the other aphids.

### S. symbiotica CWBI-2.3^T^ decreases the survival of some aphid hosts.

Next, we investigated how CWBI-2.3^T^ affected a component of aphid fitness. We monitored the survival of aphids for 7 days after colonizing them with CWBI-2.3^T^-GFP through feeding (Fig. 4C). We observed similar impacts of CWBI-2.3^T^-GFP on the survival of A. pisum and A. fabae. After 7 days, the treated A. fabae and A. pisum populations lost 15% and 19% more aphids than their respective control populations. This decrease was not significant for A. fabae and was only marginally significant for A. pisum (*P = *0.16 and *P = *0.013, log-rank tests, respectively). These results are consistent with previous studies that used wild-type CWBI-2.3^T^ (23, 30). Colonization with engineered CWBI-2.3^T^ had a larger effect on A. craccivora survival. After 7 days, 44% fewer colonized A. craccivora aphids remained alive, compared to the uncolonized aphids of this species, and this reduction was highly significant (*P = *0.00014, log-rank test). The effects of CWBI-2.3^T^ colonization on A. craccivora have not been tested before. Overall, these results show that CWBI-2.3^T^ can have a range of fitness effects on different aphid host species.

### Induction of S. symbiotica CWBI-2.3^T^ gene expression *in vivo*.

**FIG 5:**
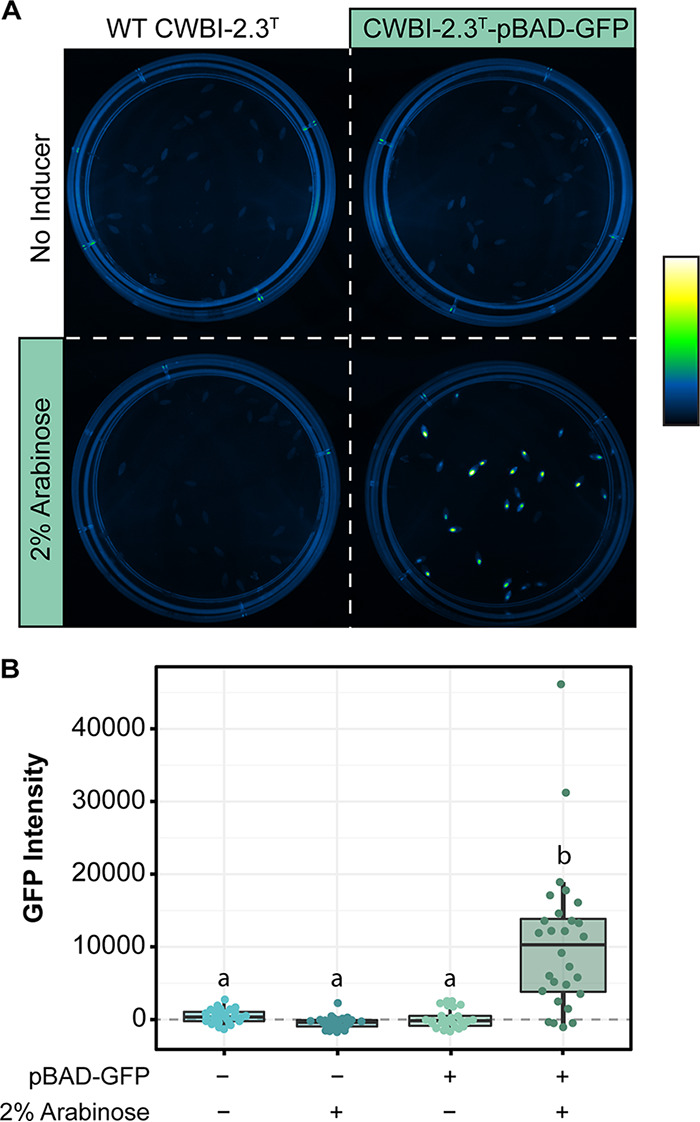
GFP induction inside living aphids. (A) Photograph of GFP expression for each of the four conditions tested for induction: CWBI-2.3^T^ with or without arabinose and CWBI-2.3^T^-pBAD-GFP with or without arabinose. The image has been pseudocolored and linearly adjusted for contrast. (B) Boxplots showing the fluorescence distribution of each population of treated aphids. The GFP intensity of at least 24 aphids was measured per condition. Lowercase letters (a and b) above the bars designate treatments for which significantly different colonization levels were observed (*P < *0.05, Dunn's test with Bonferroni correction).

To enable the controlled expression of genes inside living aphids, we tested whether we could induce the expression of GFP inside A. pisum. We first colonized A. pisum with wild-type CWBI-2.3^T^ or CWBI-2.3^T^ transformed with the plasmid pBAD-GFP (strain CWBI-2.3^T^-pBAD-GFP). Three days later, we fed the same aphids on leaves embedded in agar containing no arabinose or 2% arabinose. We observed that, after 1 day of feeding, only the aphids that were colonized with CWBI-2.3^T^-pBAD-GFP and fed on 2% arabinose showed visible GFP fluorescence (Fig. 5A). We quantified the per-aphid fluorescence under each condition and found that arabinose induction significantly increased the GFP signal in the CWBI-2.3^T^-pBAD-GFP aphids (*P = *6.9 × 10^−8^, one-tailed Mann-Whitney *U* test) (Fig. 5B). On average, GFP fluorescence was 83.5 times brighter with induction, compared to the uninduced condition.

We confirmed that the lack of fluorescence under the uninduced CWBI-2.3^T^-pBAD-GFP condition was not due to poor colonization or loss of plasmid function by crushing the aphids and plating their bacteria on medium containing 2% arabinose. We found that 93% and 100% of the aphids under the uninduced and induced CWBI-2.3^T^-pBAD-GFP conditions, respectively, were colonized with S. symbiotica. Under both conditions, the plated bacteria all expressed GFP, indicating that the plasmid had not been lost or mutated. The discrepancy between the percentage of aphids that were colonized (100%) and how many visibly fluoresced (82.8%) under the induced condition and variation in the fluorescence per aphid are likely attributable to differences in when and how much individual aphids fed on the inducer under the experimental conditions.

## DISCUSSION

In this study, we establish a platform that can be used for aphid paratransgenesis. We found that Serratia symbiotica CWBI-2.3^T^ is compatible with both broad-host-range and E. coli plasmids and gene expression parts. We fed engineered CWBI-2.3^T^ back to multiple aphid species, tracked gut colonization over time, and characterized the effects on insect survival. Lastly, we showed that an engineered function in CWBI-2.3^T^ could be induced within living aphids. This work provides new tools that can be used to increase our understanding of aphid-symbiont relationships, as well as a foundation for developing methods for the control of aphid pests without the need for insect or plant genetic engineering.

Previous studies of CWBI-2.3^T^ characterized its relationships with A. fabae and A. pisum (23, 30). These experiments established the basis for the hypothesis that CWBI-2.3^T^ and related strains may resemble a transitional protosymbiont of aphids. In particular, S. symbiotica CWBI-2.3^T^ does not colonize aphid bacteriocytes or exhibit vertical transmission to offspring (23, 25), which are important characteristics of obligate and facultative bacterial symbionts that have coevolved with aphids. Our results with engineered CWBI-2.3^T^ support the original findings in A. pisum and A. fabae, and we found similar results with two additional aphid species, A. craccivora and L. erysimi. CWBI-2.3^T^ colonizes the guts of all these aphids and can have mild to moderate negative effects on aphid fitness. These results support the hypothesis that this strain represents a transitional state of symbiosis.

S. symbiotica provides a unique opportunity to study the evolution of aphid symbioses because the entire spectrum of known host-microbe relationships is represented within a single bacterial species (28, 29). CWBI-2.3^T^ and its relatives are aphid protosymbionts and retain their ability to survive outside the host. S. symbiotica Tucson, IS, and similar strains are more specialized facultative symbionts of many aphid species. S. symbiotica SCt, SCc, and STs have evolved to act as co-obligate symbionts with B. aphidicola in Lachninae aphids. As transitional symbionts, CWBI-2.3^T^ and related S. symbiotica strains likely have genetic features that distinguish them from the more specialized endosymbiont clades. For example, the genome of CWBI-2.3^T^, while reduced, is about 1 Mb larger than that of the bacteriocyte-associated S. symbiotica Tucson strain found in A. pisum (28). The genomes of the CWBI-2.3^T^-like S. symbiotica strains encode potential pathogenicity factors that are missing in the bacteriocyte-associated strains, which could contribute to their more volatile relationships with their hosts (37).

We expect that our work will empower future studies of the protosymbiotic relationship between CWBI-2.3^T^ and aphids. By engineering CWBI-2.3^T^, we were able to observe colonization dynamics in living aphids and easily distinguish the colonization status of the aphids due to their fluorescence. This feature simplifies large assays in which colonization is required, such as mortality or transmission experiments. Similar work engineering Arsenophonus nasoniae, a parasitoid wasp symbiont, to express GFP allowed researchers to better understand how it is vertically transmitted during oviposition (38). In the future, the ability to engineer CWBI-2.3^T^ and deliver it back to aphids could be combined with engineering tools for gene knockout. This approach was used to study the role of the type III secretion system of Sodalis glossinidius in establishing a symbiotic relationship with its tsetse fly host (39). Similar genetic approaches could be used to understand the role of putative symbiotic factors in CWBI-2.3^T^ that might enable it to transition from a free-living, plant-associated bacterium to an insect symbiont.

Engineering CWBI-2.3^T^ to manipulate aphid biology could also be of interest for agricultural applications. It has been proposed that insect paratransgenesis could be used for targeted pest control strategies that pose less of a risk to the environment than chemical pesticides (9, 40). In aphids, one approach would be to engineer CWBI-2.3^T^ to reduce the capacity of aphids for vectoring plant diseases. Related techniques have already been used by groups performing paratransgenesis in other insects. For instance, Pantoea agglomerans, a symbiont of the glassy-winged sharpshooter, was engineered to produce antimicrobial peptides that selectively kill the phytopathogen Xylella fastidiosa, which is carried and spread by sharpshooters (8). Another *Serratia* symbiont, *Serratia* AS1, was engineered to limit mosquito transmission of the malaria parasite (5). These approaches took advantage of the fact that symbiont and pathogen occupied the same gut environment within their host to successfully reduce pathogen transmission. In aphids, CWBI-2.3^T^ could be used to similarly prevent the spread of bacterial phytopathogens that propagate within aphids, such as Erwinia aphidicola, Dickeya dadantii, and Pseudomonas syringae (41).

Our ability to engineer CWBI-2.3^T^ could also be adapted to enable symbiont-mediated RNA interference (RNAi) to control aphid gene expression and vectorial capacity. This method enables targeted gene silencing by engineering the symbiont to produce double-stranded RNA inside the host to induce its innate RNAi response. Symbiont-mediated RNAi has proved to be effective for genetically manipulating insects such as kissing bugs and western flower thrips, which are typically less compatible with injecting or feeding double-stranded RNA (4, 6). This approach has also recently been used to protect honeybees from parasitic mites and deformed wing virus (42). In aphids, symbiont-mediated RNAi could be used to directly target plant viruses that circulate and/or propagate in aphids (e.g., luteoviruses such as potato leaf roll virus and rhabdoviruses such as lettuce necrotic yellows virus) (43, 44). It might also be used to reduce the expression of the receptors to which noncirculative viruses bind, such as the Stylin-01 receptor bound by cauliflower mosaic virus (45).

Overall, the capabilities for engineering S. symbiotica CWBI-2.3^T^ that we have demonstrated could lead to a multitude of new applications in the future. They may help researchers build a better understanding of the evolution of host-microbe symbioses in the well-established aphid model system. These tools also provide a foundation for exploring new synthetic biology approaches for pest management that could lead to safer and more environmentally friendly agricultural practices.

## MATERIALS AND METHODS

### Growth of S. symbiotica CWBI-2.3^T^.

We obtained S. symbiotica CWBI-2.3^T^ from the German Collection of Microorganisms and Cell Cultures (DSM 23270). Bacteria were cultured at room temperature (∼25°C) in BBL trypticase soy broth (TSB) or on trypticase soy agar (TSA) plates (Becton, Dickinson and Company, MD, USA). For liquid cultures, the tubes were incubated with orbital shaking at 180 rpm over a diameter of 1 inch. Concentrations of antibiotics and other medium supplements used in this study were as follows: carbenicillin, 100 μg/ml; chloramphenicol, 20 μg/ml; gentamicin, 40 μg/ml; kanamycin, 50 μg/ml; spectinomycin, 60 μg/ml; diaminopimelic acid (DAP), 0.3 mM. To assess growth rates, 2-day-old cultures of CWBI-2.3^T^ were grown up and diluted 1:100 into fresh TSB in a 96-well plate. Ten replicates were included on the plate, which was incubated at 25°C with 6-mm-amplitude orbital shaking every 15 s in an Infinite 200 PRO plate reader (Tecan, Männedorf, Switzerland). Optical density at 600 nm (OD_600_) readings were taken every 10 min for 48 h. Growth curves were fit to a logistic model using Growthcurver (version 0.3.0) (46).

### Growth and maintenance of aphid colonies.

*Acyrthosiphon pisum* LSR1 was acquired from long-term stocks maintained in the laboratory of Nancy Moran (University of Texas at Austin). Aphis fabae was obtained from the laboratory of Thierry Hance (Université Catholique de Louvain, Belgium). Aphis craccivora and *Lipaphis erysimi* were collected in Austin, Texas, and identified by COI barcode sequencing using the LepF (5′-ATTCAACCAATCATAAAGATATTGG-3′) and LepR (5′-TAAACTTCTGGATGTCCAAAAAATCA-3′) primers (47). A. pisum, A. fabae, and A. craccivora were maintained on Broad Windsor Vicia faba plants (Mountain Valley Seed Company, UT, USA). L. erysimi was reared on *Brassica oleracea* var. Capitata (The Seed Plant, TX, USA). All colonies were maintained in cup cages on their respective plants at 20°C with a long (16-h light/8-h dark) photoperiod, in Percival I-36LLVL incubators (Perry, IA, USA).

### Transformation of S. symbiotica CWBI-2.3^T^.

S. symbiotica CWBI-2.3^T^ was made electrocompetent using a modification of a protocol for E. coli. Cultures were grown for 2 days until they reached saturation. Fifty microliters of saturated culture was then inoculated into 50 ml of TSB in a 250-ml flask and grown for about 16 h to mid-log phase (OD_600_ values of 0.4 to 0.6). Cells were then centrifuged at 4,500 × *g* for 5 min, the supernatant was discarded, and the cells were resuspended in 40 ml of 10% glycerol. This wash step was repeated four additional times. The pellet was then resuspended in 500 μl of 10% glycerol, divided into 50-μl aliquots, and frozen at −80°C. For electroporation, 2 μl plasmid was added to 50 μl of electrocompetent cells and electroporated at 2.5 V in a 0.1-cm cuvette with a Bio-Rad MicroPulser (Hercules, CA, USA). The cells were resuspended in 950 µl TSB, allowed to recover overnight (∼16 h), and then plated on TSA with selective antibiotic.

For conjugative transformation, cultures of the donor E. coli strain MFD*pir* and the recipient S. symbiotica CWBI-2.3^T^ strain were first grown to saturation. Then, 1 ml of each was centrifuged at 1,000 × *g* for 5 min. The supernatant was discarded, and the pellets were resuspended in 1 ml 145 mM saline and centrifuged again as before. The wash step was repeated, and both pellets were resuspended in 500 μl of 145 mM saline. A 50-μl sample of donor and recipient cells at a 1:100 ratio was prepared in a separate Eppendorf tube and then spot plated on a TSA plus DAP plate. After 2 days of growth on the conjugation plate, a metal loop was used to scrape up a small pellet of bacteria from each spot and place it into 1 ml of saline solution. The pellets were then centrifuged and washed twice with saline as described in the previous steps. Next, 100 μl of resuspended solution along with 100 μl of 10× concentrated solution was plated on TSA plates containing selective antibiotics. After 2 to 3 days of growth, successfully conjugated CWBI-2.3^T^ colonies were picked and screened for proper strain identity and the presence of the conjugative plasmid.

### Mini-Tn*7* integration into the CWBI-2.3^T^ chromosome.

Integration of a GFP and kanamycin resistance (Kan^r^) gene cassette into S. symbiotica to create strain CWBI-2.3^T^-GFP was carried out using the mini-Tn*7* system (32). Integration was performed according to the conjugation steps described above, with modifications for the use of two MFD*pir* donor strains. One donor strain expresses the suicide delivery vector containing the genes of interest (pTn*7*-PA1-GFP-kan), and the other holds the helper plasmid (pTNS2) containing the components of the TnsABCD site-specific transposition pathway. Conjugation of both plasmids should insert the GFP and Kan^r^ genes into the chromosome of S. symbiotica 25 bp downstream of the *glmS* gene at the *att*Tn*7* site. Chromosomal integration at the expected site was confirmed through PCR and Sanger sequencing using the GFP-mut3-forward (5′-AGCCGTGACAAACTCAAGAA-3′) and *glmS*-reverse (5′-GCCGTTGCAATTGTTGTC-3′) primers.

### Assessing plasmid origin, antibiotic resistance gene, and promoter function in CWBI-2.3^T^.

The compatibility of different origins of replication and antibiotic resistance genes was tested by transforming CWBI-2.3^T^ with various plasmids, as shown in Table 1. Transformants were picked, and plasmids were purified from cells and verified by Sanger sequencing to confirm successful transformation.

Gene expression from various synthetic promoters was tested by transforming pBTK501, pBTK503, pBTK509, and pBTK510 from the bee microbiome toolkit into CWBI-2.3^T^ (36). Each strain was grown in culture for 2 days, transferred to a fresh tube, and diluted to an OD_600_ of 0.05 in triplicate. Following an additional 1 day of growth, GFP fluorescence was measured in the Tecan Infinite 200 PRO plate reader (excitation at 485 nm and emission at 535 nm). Three technical replicates were measured for each of the three biological replicates.

To construct the pBAD-GFP plasmid, we used Gibson assembly to combine the pBR322 origin, *araC* regulator, *araBAD* promoter, and ampicillin resistance gene from the mTagBFP2-pBAD plasmid (Addgene number 54572) with the GFPmut3 gene from pBTK503 (36, 48). Overnight cultures of CWBI-2.3^T^ transformed with pBAD-GFP (CWBI-2.3^T^-pBAD-GFP) were spiked with 0.2 or 2% (wt/vol) arabinose. Three biological replicates of each condition, including the no-arabinose control, were used for this experiment. After an additional 24 h of growth, GFP fluorescence was measured using the Infinite 200 PRO plate reader (excitation at 485 nm and emission at 525 nm).

### Feeding S. symbiotica CWBI-2.3^T^ to aphids.

Feeding was carried out using age-controlled aphid populations reared on V. faba. Third instar aphids were used for all feeding experiments. Cultures of S. symbiotica CWBI-2.3^T^ were first grown to log phase, centrifuged at 1,000 × *g* for 5 min, washed twice with 1× phosphate-buffered saline (PBS), and then resuspended in 1× PBS to an OD_600_ of 1. One microliter of this culture, along with 2 μl of yellow food dye to improve aphid feeding rates (Gel Spice Company Inc., NJ, USA), was added per 100 μl of Febvay artificial diet (49). Feeding chambers were assembled using 33-mm petri dishes and two pieces of Parafilm. A hole was cut into the bottom of one half of the petri dish and covered with a piece of tightly woven mesh for airflow. A single piece of Parafilm was stretched thin over the other half, and 100 μl of diet was pipetted onto the Parafilm. The other piece of Parafilm was stretched thin over the top of the diet to create a “sandwich.” Up to 30 aphids were added to the ventilated half of the petri dish, and the two sides were sealed together with Parafilm. Aphids were fed for 16 to 24 h, transferred to V. faba plants, and observed as needed.

### Imaging aphid colonization.

Five- to 6-day-old aphids were fed on diet plates containing CWBI-2.3^T^-GFP as described above. For the time course study, A. pisum aphids were checked for colonization on the second day postfeeding using a G:Box F3 Syngene imager, and four were selected to be imaged for the time points at 2, 3, 5, and 10 days posttreatment. Between the imaging time points, the aphids were maintained on separate V. faba plants under normal conditions. For the imaging of the different aphid species at one time point, aphids were screened for colonization on day 5, and three of each were randomly selected to be imaged. A Leica MZ16 fluorescent stereoscope was used in conjunction with the Leica Application Suite software to capture all images. Images were captured in virtual stacks using both a brightfield channel and a GFP channel. To qualitatively highlight the localization of the colonizing bacteria, the brightness and contrast of the images were linearly adjusted using ImageJ (version 1.52p) as necessary.

To acquire confocal images of aphids, A. pisum aphids were fed on plain diet or diet containing CWBI-2.3^T^-GFP and were dissected at 10 days after feeding. Guts, bacteriocytes, and embryos from three colonized aphids and one control were mounted in PBS solution on glass slides for imaging. Brightfield and GFP channel images were captured on a Zeiss LSM 710 laser scanning confocal microscope, and the resulting images were processed using ImageJ.

### Quantifying colonization of aphids with CWBI-2.3^T^.

Each species of aphid was first fed on diet containing CWBI-2.3^T^-GFP at 5 to 6 days of age, as described above. Aphids were fed in pools of 25 to 30 aphids per container. After 24 h, aphids were moved to V. faba plants. The day when the aphids were transferred to plants served as day 0 for the colonization experiments. On days 2, 3, 5, and 10, aphids were collected and processed. First, aphids were surface sterilized by soaking in 200 μl 10% bleach for 1 min. Aphids were then rinsed with distilled water, resuspended in 200 μl saline, and squashed with a pestle. The squashed aphid mixture was serially diluted 10-fold to reach a final dilution of 1:10^5^. Five microliters of each dilution mixture was spotted on TSA plus kanamycin plates (three replicates per aphid). The plates were incubated for 3 days, and then colonies were counted to determine the CFU per aphid.

### Aphid fitness following colonization with CWBI-2.3^T^.

We assessed the effect of CWBI-2.3^T^ on the fitness of each aphid species by measuring mortality rates. Aphids were fed on either plain diet or diet containing CWBI-2.3^T^-GFP. Following treatment, they were transferred to plants, and the number of living adult aphids was recorded each subsequent day. To determine whether aphids that died prior to day 5 were colonized with CWBI-2.3^T^ (before it expressed sufficient GFP to be detected by eye), these dead aphids were collected, washed in bleach, squashed, and plated as described above. Pieces of mesh material were wrapped around the base of each plant at the start of the experiment so that dead aphids could be easily collected. On day 5, the remainder of the uncolonized aphids were visually sorted and removed from the experimental pool. Death was recorded for a total of 7 days posttreatment.

### Inducible control of GFP expression *in vivo*.

The assay for the function of *in vivo* induction was carried out in two separate feeding steps, namely, colonization with CWBI-2.3^T^ and induction with arabinose. To colonize the aphids with the symbiont, aphids were first fed on diet plates containing either wild-type CWBI-2.3^T^ or CWBI-2.3^T^-pBAD-GFP. After feeding on diet for 24 h, the aphids were transferred to *V. faba* plants. Induction plates were created using 2-cm-diameter petri dishes. Each dish was propped up at an angle, and 1 ml 1.5% agar with or without 2% (wt/vol) arabinose was added. V. faba leaves were added to the agar as it cooled, and plates were stored at 4°C. All induction plates were prepared 2 days before aphids fed on them. The aphids were transferred to the induction plates after resting on plants for 3 days. Three plates containing 30 aphids each were set up for each condition: CWBI-2.3^T^, CWBI-2.3^T^ plus 2% arabinose, CWBI-2.3^T^-pBAD-GFP, and CWBI-2.3^T^-pBAD-GFP plus 2% arabinose.

After 24 h of feeding on the induction plates, 30 aphids per condition were randomly selected and pooled, and GFP expression of all the aphids was photographed using the G:Box F3 Syngene imager with a blue LED excitation light and an SW06 emission filter. Plates were photographed together in one image to ensure comparable fluorescence measures. To confirm that the aphids were colonized and that the number of aphids showing fluorescence was equivalent to the number of aphids colonized with CWBI-2.3^T^-pBAD-GFP, aphids from the induced and uninduced conditions were squashed and 10-fold dilutions were plated onto TSA plus carbenicillin plus 2% (wt/vol) arabinose plates using the protocol used to quantify colonization of aphids described above.

Images were analyzed using ImageJ (version 1.52p). To measure GFP intensity, first the background was subtracted from the image using the rolling ball method. Then, image intensity was linearly adjusted to bring the background value for the plate down to zero. This background subtraction step was performed separately for each plate in the image (i.e., each set of aphids under one condition). Finally, the GFP intensity for each aphid was determined by outlining it and using the measurement tool to calculate the integrated density within this region of interest. To account for aphid autofluorescence, the average integrated density of all aphids under both wild-type CWBI-2.3^T^ conditions was subtracted from the measurements made under all four conditions.
